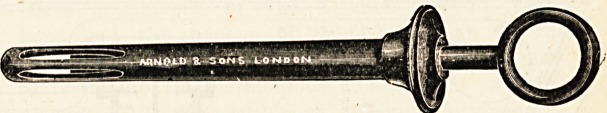# New Appliances and Things Medical

**Published:** 1901-05-11

**Authors:** 


### NEW APPLIANCES AND THINGS MEDICAL.

[We shall be glad to receive, at our Office, 28 & 29 Southampton Street, Strand, London, W.C., from the manufacturers, specimens of all new preparations and appliances which may be brought out from time to time.]

### KANNENBEER.

(KANNENBEER SUPPLY, (LONDON), LIMITED, PORTLAND ROAD, SEVEN SISTERS, LONDON, N.)

To make use of a somewhat paradoxical expression the latest stage in the evolution of the bottle is a jug. As a vessel for containing and preserving beer the ordinary bottle has certain disadvantages—firstly, owing to its shape with long narrow neck and grooved bottom it is particularly difficult to clean satisfactorily; secondly, the glass of which it is composed must necessarily be more or less transparent and pervious to light, and light rays acting for any length of time on the contained beer effect certain chemical changes which are said to materially detract from the flavour and appearance of the beer; thirdly, there are many objections to the ordinary cork as a means of sealing the bottle, and screw tops become lost and broken, and a consequent source of expense and annoyance. The Kannenbeer jug has been designed to obviate these objections, and from an examination and trial of the method we are prepared to agree with the manufacturers that the objects have been well fulfilled. The jug itself is of stoneware. It closes hermetically by a simple and ingenious lid, which is held in position by a clasp which carries the seal of the company. In structure the jug is of elegant shape, and well suited for the table; it has a large opening at the neck and a flat bottom, consequently it can be easily cleaned. The jug and handle are in one piece and made especially strong to resist ordinary wear and tear. Practical tests show that the jug is an admirable vessel for the preservation of the contained beer: the liquor invariably comes up to the table in fine condition, sparkling, and free from sediment. The beer is guaranteed best Burton brewed and free from preservatives. From a hygienic point of view this beer has much to recommend it. The beer is in the first place of fine quality, and secondly, well preserved, and this, combined with the fact that it cannot be subsequently tampered with or diluted, should popularise Kannenbeer, not only among private persons, but also among employés who are entitled to a fixed and measured amount. With all these advantages Kannenbeer is no more expensive than ordinary beer. The price of the dinner ale is 3s. per half-dozen jugs, equivalent to one dozen imperial pints. In addition to dinner ale, India pale ale, stout, half-and-half, and lager beer can be obtained jugged for use in the same convenient manner.

### NEW RECTAL OINTMENT INTRODUCER.

(ARNOLD AND SONS, 31 WEST SMITHFIELD, LONDON, E.C.)

THE above firm, acting on the suggestion of Dr. Alexander Duke, of Cheltenham, have succeeded in manufacturing a very convenient form of ointment introducer, which possesses distinct advantages over those usually employed in rectal cases. As may be seen from the illustration, the free end of the syringe is widely fenestrated by four longitudinal openings. The instrument is charged for use by withdrawing the piston and plunging the fenestrated end into the ointment to be used. The syringe is then quickly rotated and withdrawn, when it will be found filled and ready for use. The great advantages of this instrument are (1) the great simplicity, (2) the ease with which it can be perfectly cleaned and freed from ointment after use. It is made in vulcanite and can be employed for other than rectal cases.

### BOVININE.

(THE BOVININE COMPANY, 66 HATTON GARDEN, LONDON, E.C,)

BOVININE is a liquid meat juice which has stood the test of many years' clinical experience, and unlike many of the meat extracts on the market it is not a concentrated essence of the extractives of flesh which Crofton and other observers have proved to be not only of no nutritive value, but actually possessing properties most damaging to the tissues when submitted to them in concentrated form. Crofton has proved by a series of most beautiful experiments that the symptoms of gout are falsely ascribed to the action of uric acid. The various extractives of meat, which belong to the group of aloxuric bases, such as zanthin, hypozanthin, guanin, and creatinin on the other hand can cause a rapid and serious necrosis of delicate animal tissues. It is these bodies which we introduce into the system when we administer ordinary meat extracts which are soluble in boiling water. Bovinine contains no larger percentage of these injurious bases than is contained in ordinary fresh meat; soluble proteids, however, are contained in liberal percentage, thus constituting the preparation a really nutritive fluid—which has lost none of those vital properties which are so essential for the maintenance of life in conditions of malnutrition, rickets, and scurvy. What these vital properties are has long been a question of the greatest interest to physiologists and clinicians, but it seems probable that in some way they are connected with the organic combination of salts with some proteid or nitrogen-containing principle. This combination is destroyed by heat and other chemico-mechanical processes which are employed in preparing ordinary meat extracts. Bovinine possesses the same principles as freshly prepared meat juice, is always ready to hand, and requires no skill in the preparation—it has simply to be diluted with some simple fluid, which must be cold, and given to the patient.

## Figures and Tables

**Figure f1:**